# CMOS compatible 2T pixel for on-wafer in-situ EUV detection

**DOI:** 10.1186/s11671-023-03836-2

**Published:** 2023-06-20

**Authors:** Wei-Hwa Lin, Han-Lin Huang, Pin-Jiun Wu, Chrong-Jung Lin, Ya-Chin King

**Affiliations:** 1 Institute of Electronics Engineering, National Tsing Hua University, Hsinchu, Taiwan; 2 College of Semiconductor Research, National Tsing Hua University, Hsinchu, Taiwan; 3 National Synchrotron Radiation Research Center, Hsinchu, Taiwan

**Keywords:** EUV, Detector, CMOS

## Abstract

A novel 2-transistor (2T) pixel EUV detector is proposed and demonstrated by advanced CMOS technology. The proposed 2T detector also exhibits high spectral range (< 267 nm) and spatial resolution (67 μm) with high stability and CMOS Compatibility. The compact 2T EUV detector pixels arranged in a test array are capable of on-wafer recording the 2D EUV flux distribution without any external power. The compact 2T EUV detector pixels arranged in a test array are capable of on-wafer recording the 2D EUV flux distribution without any external power. Through proper initialization process, EUV induced discharging mechanism is fully investigated and an EUV induced electron emission efficiency model is established. Finally, a 2D array for in-situ EUV detection is demonstrated to precisely reflect the pattern projected on the chip/wafer surface.

## Introduction

In recent years, Extreme Ultraviolet (EUV, 10–100 nm or 13.5 nm) radiation has become one of the core technologies in modern semiconductor manufacturing industry. According to Moore’s law, it has been projected that the total number of transistors on a single chip will double every a few years [1, 2]. By scaling down the critical dimensions (CD) in ICs, the semiconductor industry continuously pushed the circuit performance and its complexity to the next stages [3, 4]. In the past decade, the mainstream lithography system adopts Deep Ultraviolet (DUV) light of wavelength from 300 to 193 nm with sources from krypton-fluoride (KrF) lasers and argon-fluoride (ArF) excimer lasers [5, 6]. Limited by its wavelength, DUV systems is unable to further reduced CD as one enters 7 nm technology node [7]. To break through this bottleneck, EUV based lithography system is proposed, enabling the transfer of patterns reliability for IV production of advanced technology node. Applying EUV (13.5 nm) light source [8, 9], the semiconductor industry is capable of mass-produce sub-10 nm integrated circuits (IC) and optimizing their overall operational speed, power consumption and circuit density aggressively. Monitoring the EUV light in the scanner chamber has become one of the key challenges to ensure the high uniformity, accuracy and stability in modern nanoelectronics manufacturing [10].

Previous studies [11, 12] have reported that EUV will be absorbed by air and thin films used in semiconductor ICs, including Cu (metal line) and SiO_2_ (Inter Layer Dielectric, ILD), the most common materials in back-end-of-line process. In addition to ensure there is no outgassing in a vacuum environment, EUV monitoring generally requires specially designed detectors. Typically, silicon-based photodiode including Schottky photodiode are used in EUV lithography system, and the EUV responsivity is obtained by measuring the photocurrent [13–15]. However, continuous degradation of EUV detectors is reported on silicon-based photodiodes [13] which cannot endure long-term bombardment of high-energy photons. Besides, most EUV photodetectors require customized fabrication process [16], increasing the complexity of integrating the EUV photodiodes with read-out circuits, essential to building sizable pixel arrays.

Conventional CMOS compatible detectors, such as Active Pixel Sensor (APS) can be applied to EUV detection [17]. APS arrays with backside illumination can maintain some EUV sensitivity. Nevertheless, they still subject to degradation after long term exposure [17]. Also, external power supply is required on these conventional arrays to operation, which makes them less approachable for the environments in lithography chambers, such as high vacuum level or under liquid immersion [18–20].

In this work, an on-wafer, in-situ 2T pixel array for EUV sensing is proposed and demonstrated. 2T pixel array with buried sensing pad is proposed and demonstrated for detecting other sources in lithography system including DUV and electron beam. In this work, the proposed detector has been modified to be applied to in EUV detection [21–23]. Featuring CMOS logic process compatibility, the proposed 2T EUV detectors are directly made on Si-wafer, and can be placed in EUV scanner directly. Unlike traditional detectors with Si-based sensing layer [24], the proposed EUV detector uses metal sensing pads. The current generated through the photoelectric effect is immune to degradation after EUV bombardment. By in-line EUV detection and off-line reading, the compact 2T EUV detector can reflect the projected light intensity without any external power module, making it safe and accessible in processing chambers without any contamination concerns [25]. By analyzing EUV induced discharging on the proposed p-channel floating gate detector, an electron emission efficiency model is established. The proposed 2T pixel is capable of precisely reflecting the 2-dimensional EUV patterns, making it a promising solution of providing on-wafer light detection, feedback module for advanced lithography system. The performance parameters of the proposed sensor and other sensors are summarized in Table 1.

**Table 1:** Performance parameters of Different Sensors

Detector	Spectral range	Spatial resolution	Detection power	Stability	CMOS compatibility
2T Detector	< 267 nm	67 μm	No need	Good	Fully compatible
Photodiode [26]	120 nm–250 nm	> 1 cm	High	Not stable	Not compatible
Photodiode [27]	< 220 nm	3.7 mm	High	Good	Not compatible
APS [28]	5 nm-1 μm	10 μm	Medium	Not stable	Highly compatible

## Pixel circuit and operation principles

**Fig. 1:**
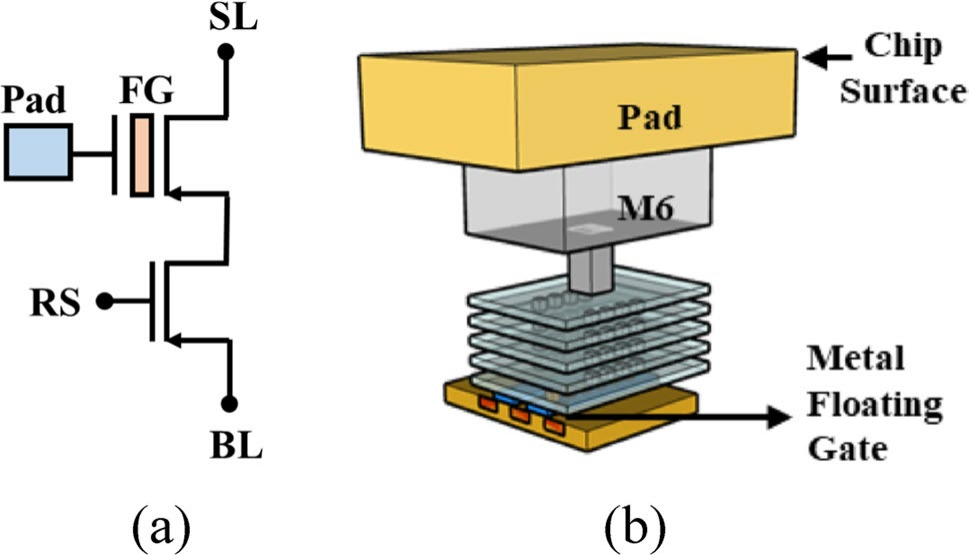
The **a** circuit schematic and **b** 3D structure of the proposed EUV detector, including 2 PMOS in series. Row select (RS) transistor is used for pixel selection, while EUV detection and data storage are achieved by the floating gate (FG) transistor in series to the select transistor

The circuit schematic of the proposed 2T pixel for EUV sensing is shown in Fig. 1a. Two p-channel Metal–Oxide–Semiconductor Field Effect Transistor (MOSFET) are placed in series to construct a single pixel. Unlike traditional floating-gate-based device, there is no extra coupling structure to control the FG potential, EUV data read-out is completed by measuring the channel current affected by the FG potential. The row-select (RS) transistor is used for pixel selection during initialization step and data read-out, while EUV detection and data storage made possible by the floating gate (FG) transistor.

**Fig. 2:**
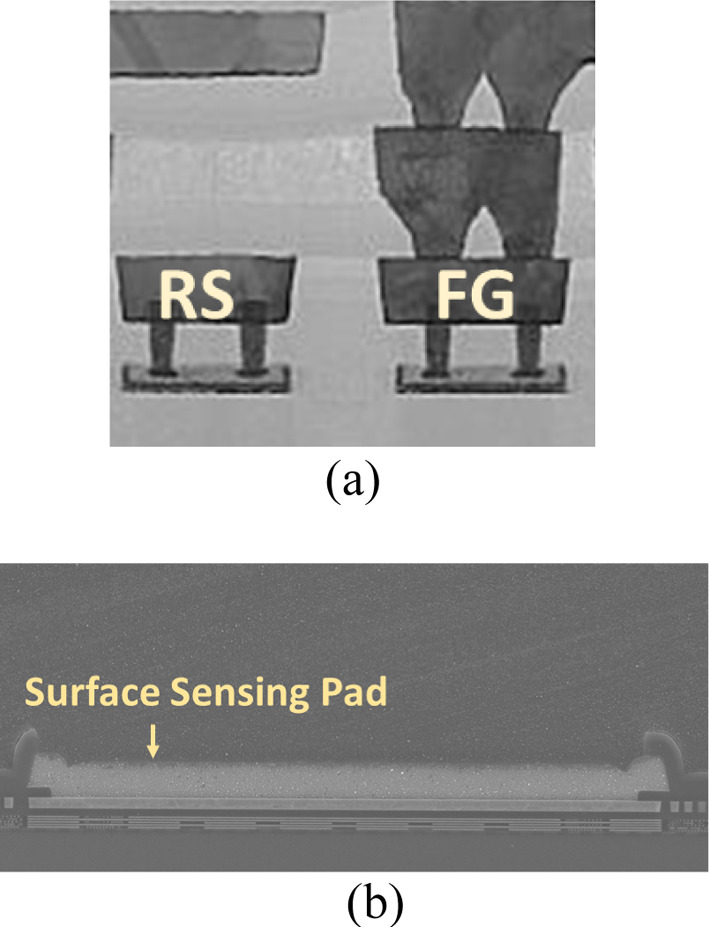
The TEM photograph of **a** the propose EUV detector and **b** SSP. The detector samples are fabricated by 28 nm CMOS logic process

**Fig. 3:**
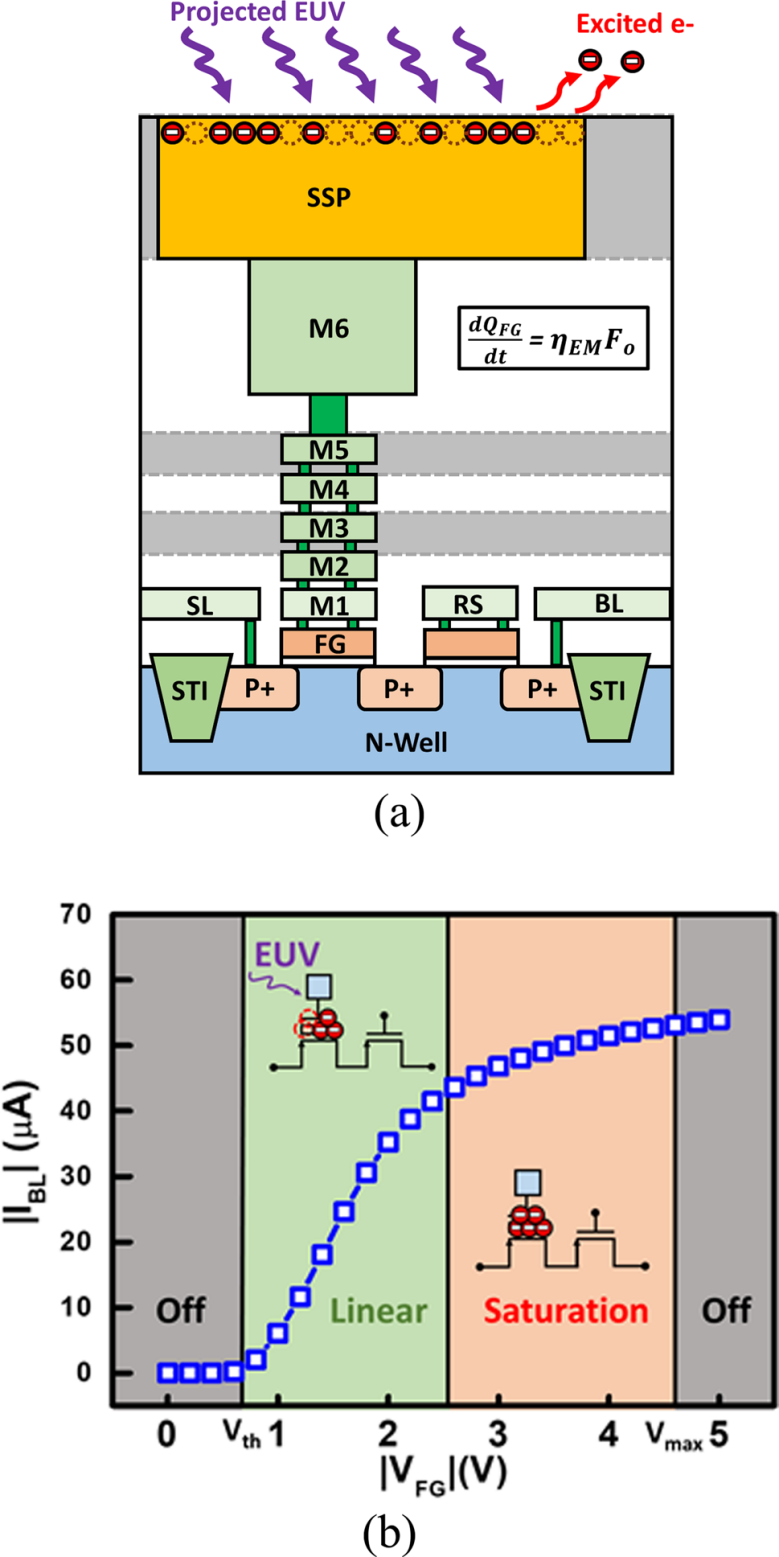
**a** Illustration of the basic operation principle of this detector **b** The corresponding FG potential at different measured BL current. The three different detector’s regions are proposed and shown in the insets

Previous studies have reported that EUV is well absorbed by dozens of materials [11, 12]; to collect the projected photons, the floating gate of the detectors are extended from metal gate to the surface pad, leading to a surface sensing node. The 3D structure of the proposed 2T EUV detector is illustrated in Fig. 1b. During EUV exposure, the high energy photons are directly projected onto the surface sensing pad (SSP) and excite the electrons stored in the FG transistor. The excited electrons with high energy will then overcome the energy barrier and escape from the floating gate, which lowers the floating gate potential. The Transmission Electron Microscope (TEM) photograph of the proposed EUV detector structure is in Fig. 2a and the cross-sectional view of the SSP is shown in Fig. 2b. During EUV exposure, the stored electrons are excited by 92 eV photons and leave the floating gate to the surface. The amount of charge-lost can reflect the EUV dosage, i.e., intensity × exposure time, as illustrated in Fig. 3a.

The relation between floating gate charge (*Q*_FG_) and EUV induced electron discharging is crucial to the following analysis. The amount of excited *Q*_FG_ can precisely reflect the EUV flux intensity. Consequently, *Q*_FG_ extraction is the first step. Here, a specially-designed dummy cell, where the FG terminal can be directly probed and measured. In addition, the gate potential can be specified, therefore, the change in channel current with respect to FG potential, *V*_FG_, can be directly observed. Based on measured BL current characteristics, three different regions can be identified as floating gate potential moves from 0 to more negative direction. In off region, the floating gate potential is lower than the threshold voltage (*V*_th_). BL current is dominated by noise when floating gate potential is lower than the threshold voltage, therefore the amount of* Q*_FG_ cannot be determined [29, 30]. If *V*_FG_ is larger than *V*_max_, FG cannot effectively retain stored charge. Therefore, this region is not suitable for EUV sensing. In the saturation region, the current is limited by RS transistor. Hence, measured the current is less sensitive to the floating gate potential in this circumstance, causing poor response toward EUV exposure. When floating gate transistor operates in the linear region, there is a linear dependency between channel current and floating gate potential, see Fig. 3b. When the proposed 2T EUV detector operates in the linear region, the detector holds the better sensitivity and is capable of reflecting the *Q*_FG_ level, hence it becomes the most desirable operation region. Before EUV exposure, it is best to raise the floating gate potential to the boundary between linear and saturation region by hot-hole induced hot electron injection (HHIHEI) [31, 32] to achieve optimal response and large sensing window.

**Fig. 4:**
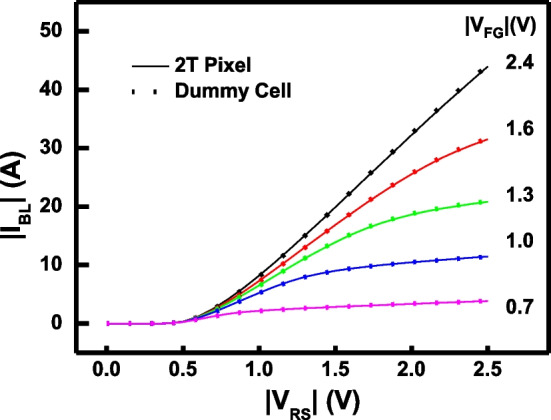
The measurement of dummy cell (symbol) and floating-gate-based 2T pixel (line) is proposed, indicating that dummy cell fitting is reliable to extract the floating gate potential

**Fig. 5:**
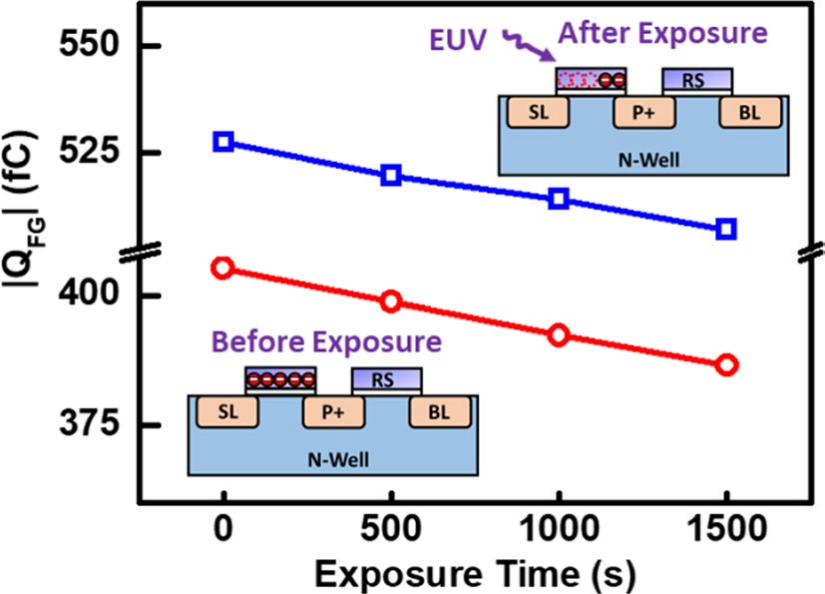
EUV discharging characteristics are compared from demonstrated two different initial states set by initialization. FG charge excited by projected photons is lost, so the total number of stored electrons will gradually decrease

As the measurement data in Figs. 4 and 5 reveals, the EUV discharging behavior is demonstrated under EUV exposure, and the amount of stored *Q*_FG_ will gradually change as the exposure time increases. Based on the previous discussion, the *V*_FG_ can be extracted by fitting the measurement date with that from a dummy cell where its gate voltage can be directly applied. Then, the floating gate charge can be obtained as follow:

$$Q_{{{\text{FG}}}} = \left( {V_{{{\text{FG}}}} - \alpha_{D} V_{D} } \right) \times C_{{{\text{FG}}}}$$

where *C*_FG_ is the total capacitance from FG terminal, and *V*_*D*_ and *α*_*D*_ are the potential and coupling ratio from the drain side of FG transistor.

From the multiplication of floating gate potential (*V*_FG_) and floating gate capacitance (*C*_FG_), the *Q*_FG_ can be obtained and the discharging rates are summarized in Fig. 5, on detectors of two distinct initial states. During EUV exposure, the EUV photons will reach the SSP and excite the stored *Q*_FG_. Subsequently, the floating gate potential and *Q*_FG_ will gradually decrease as the exposure time increases. The charge loss rate can be reflected by the measured BL current over time. Levels of Δ*Q*_FG_ is proportional to different flux intensities directly when exposure time are kept constant.

## Experimental results and discussion

**Fig. 6:**
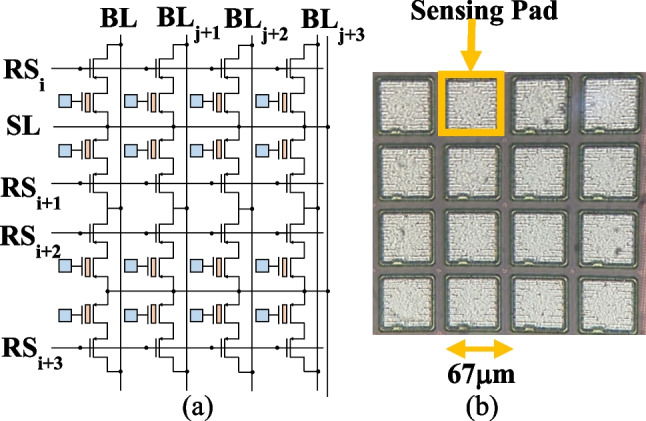
**a** The circuit schematic of 4 × 4 2T pixel array and **b** the top view photograph from optical microscope, where the white square is sensing pad used for photon collection

EUV discharging characteristic is collected from an EUV detector array, as shown on the circuit schematic of 4 × 4 pixel array in Fig. 6a, pixels are placed in a 2D array, where each pixel can be independently read on BLs sequentially [33, 34]. The top view of the 4 × 4 array from optical microscope (OM) is in Fig. 6b, where each white squares represents a surface sensing pads (SSP) with a pixel pith of 67 μm.

**Fig. 7:**
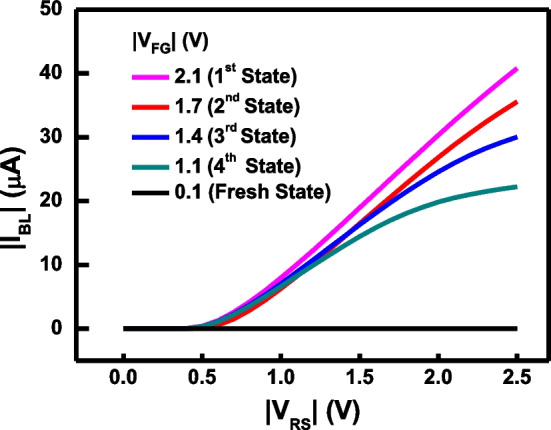
Before EUV exposure, the floating gate potential of a 4 × 4 detector array is initialized to 4 initial states by different amount of hot electron injection

The measured I-V characteristics of the proposed 2T EUV detector are compared in Fig. 7, where the initialization step can place different amount of *Q*_FG_, leading to different *V*_FG_, as indicated. For a pixel in its fresh state, due to the absence of electrons in floating gate, floating gate transistor cannot be fully turned on, and thus the EUV 2T detector is typically stay in the off-state. By channel hot carrier injection, hot electrons are injected into the floating gate, which raising *V*_FG_. By tuning the injection condition, the floating gate potential can be moved and positioned to the ideal levels, forcing the 2T detector to start at the linear/saturation region boundary, see Fig. 3b.

**Fig. 8:**
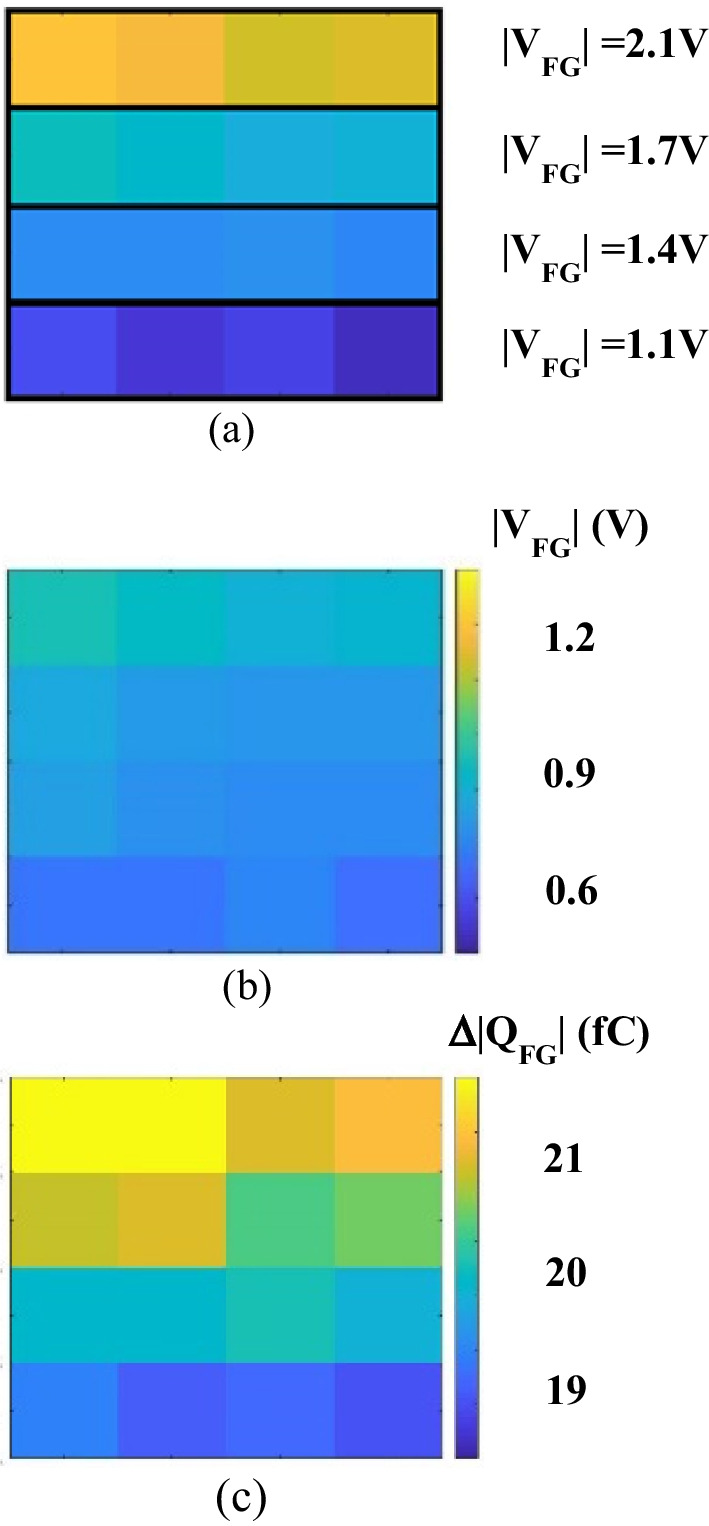
The response of 4 × 4 sensor array mapping including the FG potential **a** before, **b** after EUV exposure are demonstrated, each row is placed to different initial state; the floating gate charge shift is in **c**

To investigate the discharging characteristics at different potential level, the floating gate potential of detectors on each row is initialized to different initial states ranging from 1.1 to 2.1 V. As the measured data in Fig. 8a shows, each row is programed to one initial state to verify the relation between initial *V*_FG_ and its corresponding sensitivity. After 4.2 μW/cm^2^ 13.5 nm-EUV light exposure, the resulting *V*_FG_ and the shift of *Q*_FG_ are arranged in Fig. 8b and c respectively. As expected, *V*_FG_ becomes lower than initial state, indicate substantial amount of the electrons stored in floating gate has been excited by 13.5 nm photons and escaped from SSP. Furthermore, data in Fig. 8c reveals that under the same EUV exposure, pixels with higher initial *V*_FG_ are more responsive, namely, that more stored electrons are lost.

**Fig. 9:**
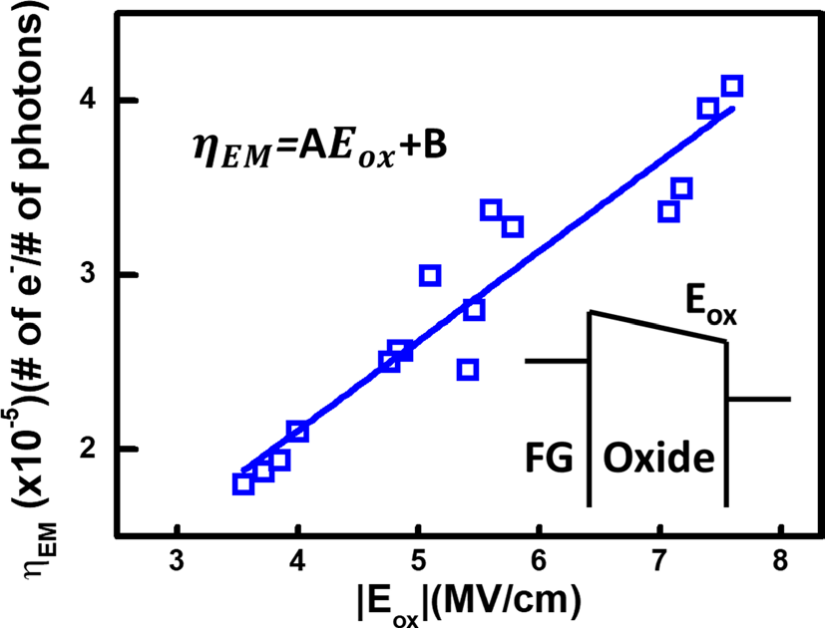
The sensitivity of the proposed 2T pixel detector can be observed, indicating a positive correlation between initial FG potential and EUV sensitivity

**Fig. 10:**
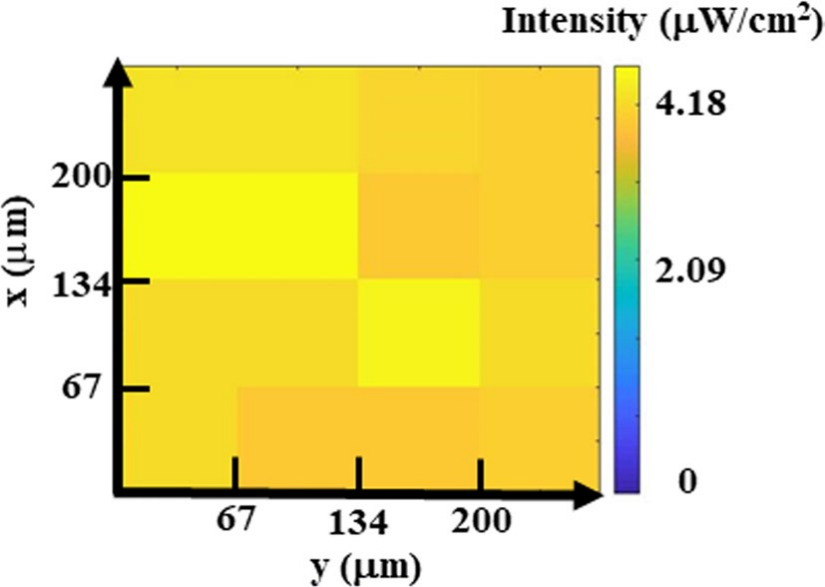
After calibration, a uniform mapping of EUV flux distribution is obtained, proving that the proposed calibration model is a promising solution for EUV detection. This data suggests that through the*η*_EM_ model, one can obtain 2D EUV distribution on a 2T pixel array

Here, the sensitivities of individual detectors evaluated and compared in Fig. 9, indicates a positive correlation between initial floating gate potential and responsivity. Under fixed EUV flux intensity (*F*_*o*_), a linear dependency between the electric field across the gate oxide (*E*_ox_) and the emission efficiency (*η*_EM_) is found as follow [21]:

$$\eta_{{{\text{EM}}}} \left( {E_{{\text{ox }}} } \right) = AE_{{\text{ox }}} + B$$

where the fitted parameters are found to be *A* = 0.1617 (cm/MV), *B* = 7.18 × 10^–4^ (a.u.). Data also suggests that higher *V*_FG_ and *E*_ox_ result in higher emission efficiency and hence sensitivity, so the floating gate potential of the proposed EUV sensor is programmed to the edge between linear and saturation region, as indicated in Fig. 3, the best sensitivity and the largest sensing window can be obtained. According to Fig. 8c, a non-uniform mapping of *Q*_FG_ shift is found under uniform EUV light. Considering the *E*_ox_-dependent emission model, the EUV distribution in the light field of 258 μm × 258 μm intensity can be more truthfully reflected, as shown in Fig. 10.

## Conclusions

In this study, a novel in-line EUV 2T detector featuring fully CMOS logic process compatibility, non-volatile data storage and high stability is proposed. The proposed 2T detector also exhibits high spectral range (< 267 nm) and spatial resolution (67 μm). Detection through proposed EUV induced discharging characteristic has also been studied comprehensively. With novel emission efficiency model, the proposed EUV 2T detector can accurately reflect the projected EUV flux intensity on the chip/wafer surface, for application in advanced lithography systems.

## Data Availability

Not applicable.
